# Resveratrol Enhances the Cytotoxic Activity of Lymphocytes from Menopausal Women

**DOI:** 10.3390/antiox10121914

**Published:** 2021-11-29

**Authors:** Andrea Di Credico, Giulia Gaggi, Pascal Izzicupo, Ines Bucci, Angela Di Baldassarre

**Affiliations:** 1 Department of Medicine and Aging Sciences, University “G. D’Annunzio” of Chieti-Pescara, 66100 Chieti, Italy; andrea.dicredico@unich.it (A.D.C.); izzicupo@unich.it (P.I.); ines.bucci@unich.it (I.B.); 2 Beth Israel Deaconess Medical Center, Harvard Medical School Initiative for RNA Medicine, Harvard Medical School, Boston, MA 02115, USA; ggaggi@bidmc.harvard.edu

**Keywords:** resveratrol, antioxidant effects, nutraceuticals, immunomodulatory, cell-mediated cytotoxicity, noncommunicable diseases, cancer, prevention, immune system, menopause

## Abstract

Nutraceuticals and functional foods are the main sources of antioxidants and have positive effects on health through regulation of the redox balance. Accordingly, they represent a useful nutritional source for the prevention of noncommunicable diseases (NCDs). Menopausal women have an increased risk of developing NCDs due to hormonal dysregulation and the ongoing aging process. Accordingly, a healthy lifestyle and good nutritional habits are of utmost importance in this population. Resveratrol (RSV) is a natural polyphenol, and it is used as a nutraceutical given its estrogenic, anti-inflammatory, and antioxidant properties. The aim of this study was to analyze the effects of RSV on the lymphocyte cytotoxicity in menopausal women. Lymphocytes from 13 healthy menopausal women (56.18 ± 4.24 years) were isolated, and then cocultured with hTERT-HME1, a breast cell line with a precancerous phenotype. The results showed that, when treated with RSV, lymphocytes significantly increased the TNF-α production (*p* < 0.001), the formation of immune synapses (*p* = 0.009), and the target cell lysis (*p* = 0.002). No effects were detected in the lymphocyte total antioxidant capacity. In conclusion, RSV might enhance the immune surveillance in menopausal women by increasing the cytotoxic activity of lymphocytes.

## 1. Introduction

Noncommunicable diseases (NCDs) such as type 2 diabetes mellitus, cardiovascular diseases, cancer, and obesity represent the major cause of death worldwide [1]. The main risk factors include physical inactivity, tobacco and alcohol use, and poor diet [2]. Regarding the latter, nutraceuticals and functional foods represent two fundamental tools in NCDs prevention [3]. Nutraceuticals are a class of natural compounds isolated from foods that usually are available in medicinal forms (e.g., powders and pills), while functional foods are consumed as part of the usual diet and have positive effects on health, beyond their nutritional properties [4]. It has been demonstrated that both these nutritional sources are useful in NCDs prevention because of their antioxidant and immunomodulatory effects [5]. Indeed, reactive oxygen species (ROS) have a pivotal role in the development of different NCDs, promoting atherosclerotic plaque formation [6] and the onset of insulin resistance and diabetes [7]. ROS can also act on the cancer cell metabolism, allowing the adaptation of malignant cells to hypoxic and nutritional stresses [8]. Then, an optimal oxidative balance is critical for both adequate immunological function, and for NCDs prevention [9,10].

Resveratrol (RSV) is a natural polyphenol found in foods such as grapes, peanuts, strawberries, and several other plants, and it is used as a nutraceutical [11]. It has already been reported that this compound can act as an antioxidant and antimutagen agent and it exerts neuroprotective, cardioprotective, and immunomodulatory effects [12,13,14,15]. Its low molecular weight allows a rapid diffusion across cell membranes and an easy spreading into intracellular sites [16]. However, the molecular mechanisms elicited by RSV are not fully understood.

Menopause increases the risk of NCDs in women [17,18] and several efforts have been made to counteract its adverse effects [19,20,21,22]. The substantial reduction of estrogen and progesterone that characterizes menopause determines modifications of the immune system response, particularly in terms of cytotoxic activity, which can result in a higher incidence of infectious and chronic diseases [23]. As the risk of developing cancer increases with age, women going through menopause generally have a greater chance of developing cancer. Therefore, tailored physical exercise and optimal nutrition are two key factors to prevent the onset of NCDs in menopausal women [24,25,26,27,28,29].

In this study, we hypothesized that RSV could improve the antioxidant capacity and cytotoxic activity in menopausal women. For this purpose, the effects of RSV on the activation and on the effector functions of cytotoxic cells from a sample of menopausal women were evaluated.

## 2. Materials and Methods

### 2.1. Study Population and Experimental Design

**Figure 1:**
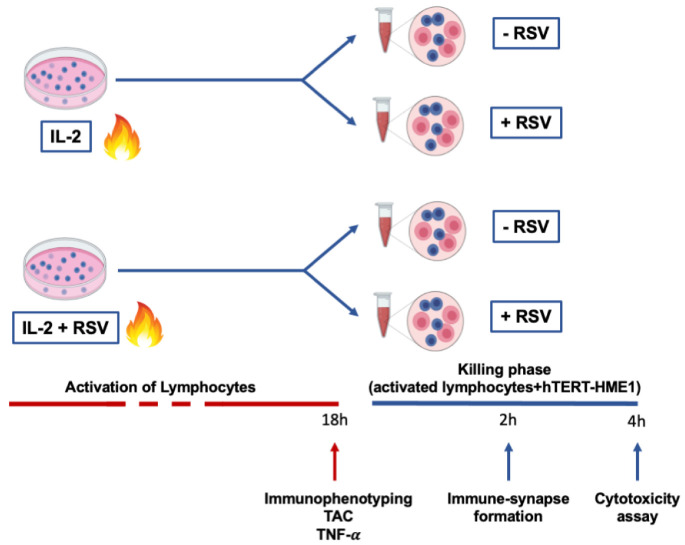
Experimental design. Lymphocytes were activated by IL-2 treatment for 18 h. At the end of the activation phase, immunophenotyping, TAC, and TNF-α production were evaluated. To analyze the effector phase, cells were then co-cultured with premalignant hTERT-HME1 cells for 4 h, and both immune synapse formation and cytotoxicity were analyzed. The blue cells in the figure represent lymphocytes, while the pink cells refer to hTERT-HME1.

This study was approved by the Ethical Committee of Chieti-Pescara University. Thirteen healthy menopausal women (56.18 ± 4.24 years) were enrolled in the study. The inclusion criteria were age <65 years; menses had naturally ceased for at least 12 months; the plasma estradiol level was <20 pg/mL; body mass index (BMI) was >18.5 and <30 kg/m^2^; no estrogen-replacement therapy or drugs were being undertaken. A 12 h overnight fasting blood sample was drawn for lymphocyte separation. Lymphocytes were activated with IL-2 in the presence or absence of RSV, and cellular immunophenotyping, total antioxidant capacity (TAC), and tumor necrosis factor-α (TNF-α) production were analyzed. Then, to test the RSV effects on the cytotoxic activity, pre-activated lymphocytes (effector cells) were co-cultured with hTERT-HME1, a premalignant model of breast epithelial cells (target cells); the immune synapse formation and lysis of the target cells were evaluated. In Figure 1, a graphical representation of the experimental design is reported.

### 2.2. Cell Cultures

The peripheral blood mononuclear cells (PBMCs) were isolated from the blood samples by Histopaque (1.077 g/mL, Sigma, St. Louis, MO, USA). Macrophages were depleted by adherence for 1.5 h at 37 °C/5% CO_2_ and non-adherent cells were cultured in RPMI 1640 (without red phenol for the TAC assay, see below; Thermofisher) 10% fetal bovine serum (FBS, Corning, Manassas, VA, USA), 2 mM glutamine, and 1% penicillin/streptomycin. For lymphocyte activation, 6 × 10^5^ cells were exposed to IL-2 (Peprotech, 200-02, 300 U/mL) for 18 h in presence or absence of 10 µM of RSV (Stemcell Technologies).

Mammary epithelial cells immortalized with hTERT (hTERT-HME1) were purchased from American Type Culture Collection (ATCC, Manassas, VA, USA) and cultured in mammary epithelial cell basal medium (MEBM, ATCC, Manassas, VA, USA) supplemented with a Mammary Epithelial Cell Growth Kit (ATCC, Manassas, VA, USA) at 37 °C/5% CO_2_.

### 2.3. Immunophenotyping Analysis by Flow Cytometry

After activation, the lymphocytes were washed twice with cold PBS and incubated with BD Multitest™ 6-color TBNK 50 Tests (644611) containing anti-CD45, anti-CD19, anti-CD3, anti-CD4, anti-CD8, anti-CD56, anti-CD16, or the appropriate isotype controls as previously described [30]. Cytometric analyses were performed with a Cytoflex cytometer (Beckman Coulter, Brea, CA, USA), and the data were analyzed with CytExpert Acquisition and Analysis Software (Beckman Coulter, Brea, CA, USA). Unstimulated cells represented the control.

### 2.4. Total Antioxidant Capacity Assay

The antioxidant capacity of the cell lysates and supernatants from activated lymphocytes was measured by a Total Antioxidant Capacity (TAC) Kit assay (ab65329, Abcam, London, UK). Briefly, lymphocytes were cultured as described above in RPMI 1640 w/o red phenol (ThermoFisher, Waltham, MA, USA) and activated with IL-2 in the absence or presence of RSV. After 18 h, 15 × 10^4^ cells were lysed in ddH_2_O, 0.05% Triton-X. Cell lysates and supernatants underwent TAC assay following the manufacturer’s instruction. The measure outputs were read with Varioskan LUX Multimode Microplate Reader (ThermoFisher).

### 2.5. Measures of TNF-α Production

TNF-α released in the cell media by activated lymphocytes was measured using a Human TNF-α ELISA Kit (KHC3011, Invitrogen, Waltham, MA, USA). The absorbance was read at 450 nm using a Varioskan LUX Multimode Microplate Reader (ThermoFisher). The cellular medium from unstimulated cells was used as control.

### 2.6. Immune Synapse Formation Assay

For the analysis of immune synapse formation, activated lymphocytes pre-stained with ViaFluor^®^ 405 (1:1000 dilution, Biotium, Fremont, CA, USA, 30068) and hTERT-HME1 pre-stained with CellTrace™ CFSE Cell (1:1000 dilution, ThermoFisher, C34554) were co-cultured at a 1:1 ratio for 2 h. Cell conjugates were then transferred to poly-L-lysin-coated coverslips. The interaction between lymphocytes and hTERT-HME1 was determined by staining the actin cytoskeleton polymerization with Rhodamine Phalloidin Reagent (1:1000 dilution, Abcam, ab235138), which highlights the F-actin at the immune synapse level. Images were acquired with a Zeiss Axio Vert. A1 inverted microscope, equipped with an Axiocam Camera 503 Mono and analyzed with ZEN Software (Carl Zeiss, Jena, Germany).

The immune synapse formation was quantified as previously reported [31]. Briefly, three different fields of each sample were analyzed, and the number of lymphocytes linked to hTERT-HME1 were quantified; the percentage of synapse formation was calculated as follows:

(1) $$\;\%\;of\;synapse\;formation=\;\frac{number\;of\;synapses}{numer\;of\;synapses\;+number\;of\;lymphocytes\;alone}\;\times \;100$$

### 2.7. Cytotoxicity Assay

To evaluate the cytotoxic effect, activated lymphocytes were co-cultured with CFSE-labeled hTERT-HME1 at a 1:1 ratio for 4 h. The cells were then stained with SYTOX™ Red Dead Cell Stain (1:1000, ThermoFisher) and analyzed by flow cytometry (Cytoflex cytometer, Beckman Coulter). The cytotoxic activity was quantified evaluating the double-positive cells (SYTOX Red^+^/CFSE^+^).

### 2.8. Statistical Analysis

A one-way analysis of variance (ANOVA) and Tukey’s post-hoc test were performed. The results were considered significant when the *p*-value was < 0.05. All statistical analysis were performed using GraphPad Prism 9.2.

## 3. Results

### 3.1. Effects of RSV Treatment on the Immunophenotyping of Lymphocytes Following the Activation Phase

**Figure 2:**
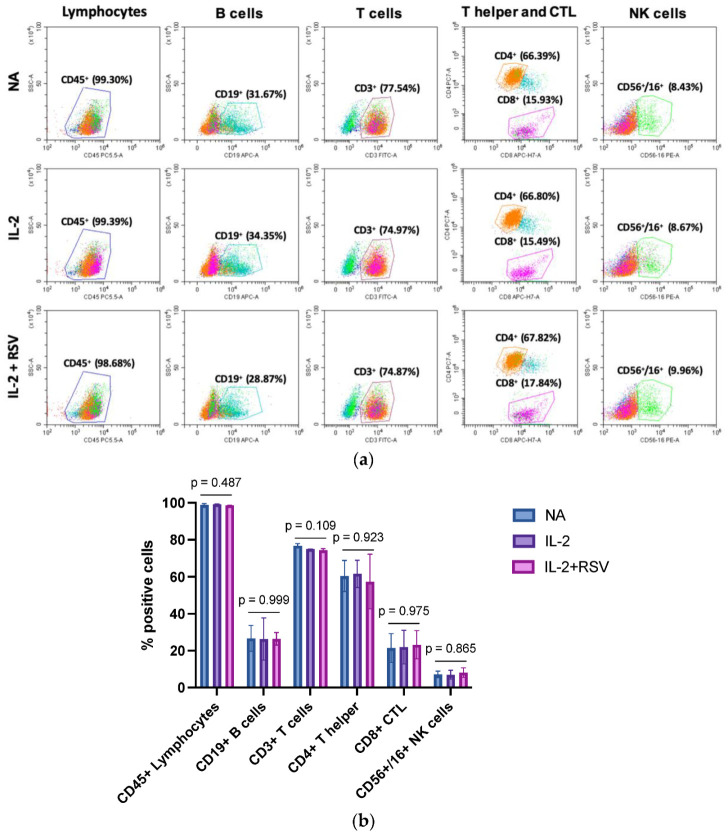
Effect of RSV on the lymphocyte populations. Lymphocytes obtained from 13 menopausal women were activated with IL-2 in absence or presence of RSV. Non-activated lymphocytes (NA) represented an internal control. (**a**) Flow cytometry graphs representative of the 13 different experiments. (**b**) Percentage of the different lymphocytic subpopulations. Data are expressed as mean ± standard deviation. CTL = cytotoxic T lymphocytes; NA = not activated; IL-2 = activated with IL-2 only; IL-2 + RSV = activated with IL-2 and RSV. The *p*-values represent the ANOVA results.

IL-2 is a cytokine required for T cell and NK activation. Once activated, lymphocytes proliferate, enhance their functions, and secrete cytokines such as interferon gamma (IFN-γ), TNF-α, and others. To evaluate a possible effect of RSV in the activation phase, we first analyzed the lymphocyte subpopulations in those samples activated with IL-2 in the absence or presence of RSV. The cells were stained with a cocktail of antibodies for CD45 (lymphocytes), CD19 (B cells), CD3 (T cells), CD4 (T helper cells), CD8 (T cytotoxic), and CD56/CD16 (NK cells) detection. The immunophenotyping analysis did not show different lymphocyte subpopulations in the activated samples in absence or presence of RSV (Figure 2).

### 3.2. Total Antioxidant Capacity of Lymphocytes after the Activation Phase

**Figure 3:**
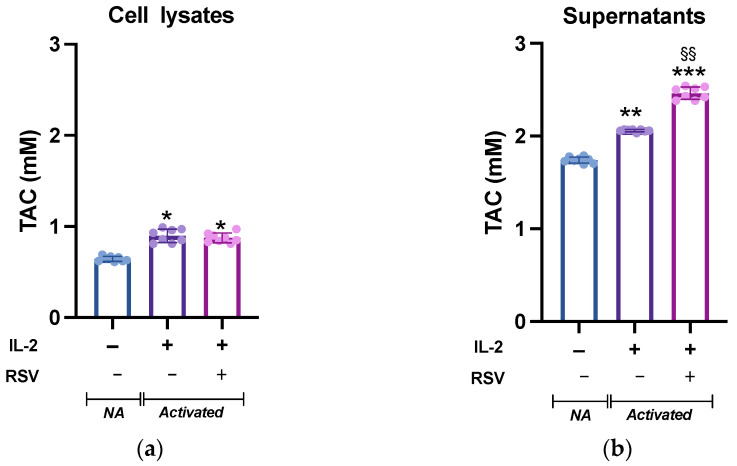
TAC levels in (**a**) lymphocytes and (**b**) supernatants from NA, IL-2-, and IL-2 + RSV- treated cells. Data are expressed as mean ± SD of Trolox equivalents (mM). NA = non-activated. * *p* < 0.05 vs. NA; ** *p* < 0.01 vs. NA; *** *p* < 0.001 vs. NA; §§ *p* < 0.01 vs. IL-2 only.

Leukocyte activation is accompanied by a dramatic increase in the metabolic demand, which results in an increased ROS production. Although ROS are critical mediators of lymphocyte signaling, high levels of ROS can be detrimental to cell survival. To avoid cellular damages caused by uncontrolled ROS production, an appropriate antioxidant capacity must be maintained to ensure the intracellular ROS levels remain at appropriate levels [32]. We then tested whether RSV could influence the lymphocyte antioxidant system. To analyze the intracellular response of the antioxidant mechanisms following RSV treatment, lymphocytes activated in absence or presence of RSV were lysed and the total antioxidant capacity (TAC; the cumulative effect of all of the antioxidants on the sample) was measured. The cell lysates from the different experimental conditions showed a significant difference (*p* = 0.019, Figure 3a). As expected, IL-2 treatment boosted the antioxidant system, and the TAC of non-activated (NA) cells was significantly lower than that of activated cells; however, no differences were found between the IL-2 and IL-2 + RSV treatments (*p* = 0.54). As the TAC of the cell medium is an important factor for the cell redox homeostasis, we complete the analysis testing the TAC in the supernatants. TAC was higher in the supernatants obtained from activated lymphocytes (1.7 ± 0.01 mM vs. 2.1 ± 0.01 mM vs. 2.5 ± 0.05, NA, IL-2, IL-2 + RSV, respectively), as observed in the cell lysates. As expected, the supernatants deriving from the IL-2 + RSV condition showed a greater TAC than that from the IL-2 condition (*p* = 0.002), probably due to the RSV itself (Figure 3b).

### 3.3. Lymphocytes’ TNF-α Release

Lymphocyte activation is a finely regulated cascade of events that results in cytokine production and secretion. To analyze whether RSV could affect the release of cytokines from activated lymphocytes, we measured the TNF-α levels in the cell media.

**Figure 4:**
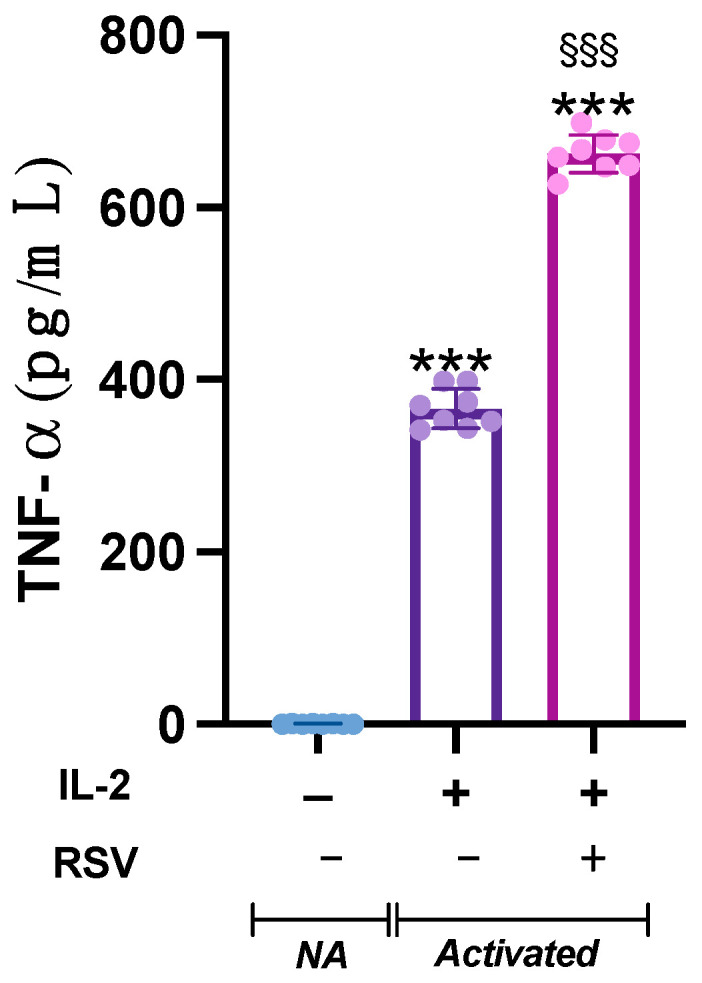
Effect of RSV on TNF-α release. TNF-α levels were measured in the supernatant from the NA, IL-2, and IL-2 + RSV samples. Data are expressed as mean ± SD. *** *p* < 0.001 vs. NA; §§§ *p* < 0.001 vs. IL-2 only.

The analysis of variance showed a significant difference in TNF-α concentration in the supernatants from the different samples (*p* < 0.001). Indeed, TNF-α, barely detectable in the NA samples, was heavily released by activated cells. Interestingly, when activated with IL-2 + RSV, lymphocytes released significant higher levels of TNF-α compared to IL-2 only (*p* < 0.001) (Figure 4).

### 3.4. Effects of RSV on the Lymphocyte Cytotoxic Activity

**Figure 5:**
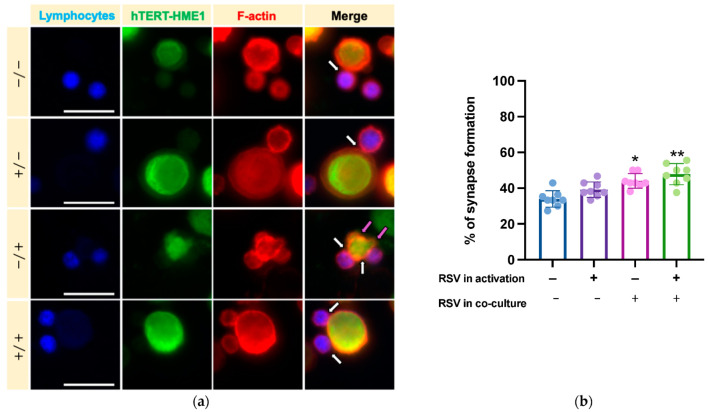
Effects of RSV on immune synapse formation. (**a**) To track the synapse formation, lymphocytes (effector cells) were pre-stained with ViaFluor (blue), hTERT-HME1 (target cells) with CFSE (green), while F-actin was evidenced with rhodamine phalloidin (red). White arrows highlight the interactions between effector and target cells, while magenta arrows indicate the blebbing of hTERT-HME1 due to cytotoxic activity. (**b**) Histograms represent the mean values and standard deviations of the percentage of synapse formation in the four different conditions. −/− = no RSV in the activation and killing phases; +/− = RSV in the activation phase only; −/+ = RSV in the killing phase only; +/+ = RSV in the activation and killing phases. Images are representative of fields from 8 independent experiments. Original magnification: 40×; scale bar = 20 µm. (**b**) Percentage of immune synapses in the different experimental conditions. Data are expressed as mean ± SD. * *p* < 0.05 vs. NA; ** *p* < 0.01 vs. NA.

We then analyzed whether RSV could influence the lymphocyte cytotoxic activity on target cells. After activation by IL-2 or IL-2 + RSV, lymphocytes (effector cells) were co-cultured with hTERT-HME1, a premalignant model of breast epithelial cells (target cells); the cells were then allowed to interact in absence or presence of RSV (Figure 1). As cytotoxic lymphocytes may kill target cells by direct cell–cell contact between effector and target cells, the cytotoxic effect was evaluated by analyzing the immune synapse formation and lysis of the target cells (killing phase). To track the immune synapse formation by fluorescence microscopy, we performed a mixed culture consisting in activated lymphocytes pre-stained with ViaFluor, and hTERT-HME1 pre-stained with CFSE (1:1 effector to target ratio) for 2 h. The samples were then treated with rhodamine phalloidin, a highly selective peptide used for staining of F-actin filaments; indeed, F-actin accumulation is the first critical cytoskeletal reorganization event occurring at the immune synapse level. The fluorescence microscopy analysis highlighted that the percentage of immune synapse formation was significantly affected by the presence of RSV in the killing phase and that the higher percentage of immune synapses was observed when RSV was present in both the activation and killing phases (Figure 5).

**Figure 6:**
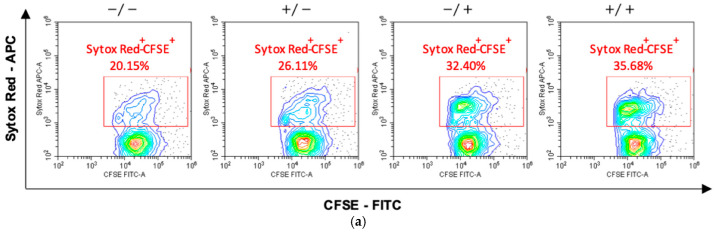

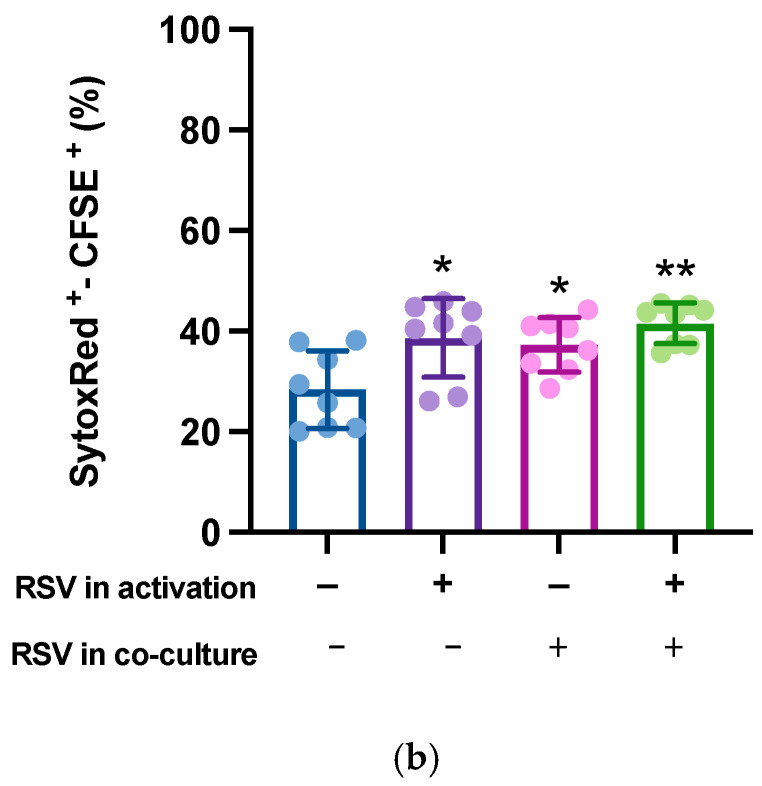
Effects of RSV on the lymphocytic killing activity on target cells. Activated lymphocytes (effector cells) were mixed with CFSE-stained hTERT-HME1 (target cells). Samples were then stained with SYTOX Red, which penetrates cells with compromised plasma membranes (specifically, it counterstains impaired cells). To quantify the killing activity, the double positive (SYTOX Red^+^/CFSE^+^) cells were analyzed. (**a**) Flow cytometry analysis representative of 8 different experiments. −/− = no RSV in the activation and killing phases; +/− = RSV in the activation phase only; −/+ = RSV in the killing phase only; +/+ = RSV in the activation and killing phases. (**b**) Percentage of compromised hTERT-HME1 cells (SYTOX Red^+^/CFSE^+^ cells) in the different experimental conditions. Data are expressed as mean ± standard deviation. * *p* < 0.05 vs. −/−; ** *p* < 0.01 vs. −/−.

Finally, to quantify the killing activity on the target cells, activated lymphocytes were mixed with CFSE-labeled hTERT-HME1 (1:1 effector to target ratio) for 4 h; compromised cells were then counterstained by specific staining (SYTOX Red) and the double-positive SYTOX Red^+^/CFSE^+^ hTERT-HME1 cells were analyzed by cytometry. The results showed that RSV significantly enhanced the lymphocyte cytotoxic activity, and that its effect was stronger when present in both the activation and killing phases (Figure 6).

## 4. Discussion

Optimal oxidative status and immune system response are key features for health. Different studies have shown that RSV, a potent antioxidant, exerts positive effects on the prevention of different diseases, particularly NCDs [33,34]. Our data highlight that RSV increases the cytotoxic activity of lymphocytes from menopausal women and suggest that RSV may play a support action in the immune surveillance against tumor cells in menopausal women.

Lymphocyte activation needs a high amount of energy and metabolites to induce clonal expansion and to develop effector functions [35,36]. However, these biological activities determine the production of ROS, which are critical second messengers for lymphocyte activation, but that can be detrimental by damaging DNA and other subcellular structures [9,10]. Therefore, a strict regulation of ROS levels by antioxidant pathways is crucial for a proper signal transduction without jeopardizing the cell integrity [37]. A proper redox balance can be maintained through adequate levels of physical activity and a healthy diet [38,39,40,41]. The antioxidant properties of RSV have already been described: RSV exerts its effects by scavenging free radicals and inhibiting ROS production [42]. It may also block the cell cycle of cancer cells, downregulating tumor-derived nitric oxide synthase [43]. RSV also exhibits some estrogenic activities [44]. Despite these well-known contributions to the cellular redox balance, we did not find differences in TAC activity in lymphocytes activated in the presence of RSV. Indeed, as expected, we registered an increase in the antioxidant properties in both lysates and supernatants obtained from activated cells [10]; however, an increment in the intracellular TAC was not further fostered by RSV. This finding could not be ascribed to possible differences in the lymphocyte subpopulations in the IL-2 or IL-2 + RSV samples; indeed, in accordance with the results from other labs [45], the lymphocyte subpopulations were equally represented in the IL-2 or IL-2 + RSV samples.

CD8^+^ T and natural killer (NK) cells are both cytotoxic effector cells of the immune system, but their biological characteristics are drastically different. Unlike T cells, NK cells can recognize and lyse the infected or transformed cells in an MHC-unrestricted manner, without previous sensitization [46,47,48], thus representing the main effector cells toward cancer [46,49,50]. As the antigen-specific killing activity of the T cytotoxic cells requires a complex and fine-tuned interaction with other cells, while NK cells display an intrinsic ability to lyse tumor cells [51], it is reasonable that the cytotoxic activity measured in our in vitro tests should be ascribed mainly to NK cells.

Lymphocyte activation also implies the secretion of specific lymphokines that regulate immune cell functions. Once activated by IL-2, NK cells enhance their cytotoxic activity, becoming able to recognize a broad spectrum of targets and to kill with enhanced cytolytic potential. Additionally, IL-2 drives the secretion of TNF-α, IFN-γ, and other important cytokines for the immunological response [52]. Recently, it has been described that RSV stimulates IFN-γ secretion from NK cells [45]; similarly, our data evidenced that the addition of RSV in the activation phase supports the lymphocyte secretion of TNF-α. It is worth noting that TNF-α enhances NK cell cytotoxicity and is considered an integral component of NK cytolytic function [53]. As IFN-γ and TNF-α act synergistically in supporting the NK cytolytic activity [54], the finding of an increased TNF-α production in IL-2 + RSV-activated samples suggests that RSV may optimize NK cell-mediated immunity, thus enhancing the immune surveillance.

Efficient cytolytic activity requires the formation of immune synapses, a tight adherence between the cytotoxic lymphocyte and the target cell; through this contact, the lytic granules are released into the target cell, where they exert their cytolytic effects [55]. Our data showed that RSV increases the immune synapse formation between cytotoxic and target cells; interestingly, the highest number of immune synapses was detected when RSV was present in both the activation and killing phases. The hypothesis that RSV could sustain the lymphocyte cytotoxic activity was further supported by the cytometric analysis. Indeed, the percentage of compromised target cells was higher in samples containing RSV, and as for immune synapse formation, the highest killing efficacy was observed when RSV was present in both the activation and killing phases. The mechanisms through which RSV can support the cytotoxic immune response are still largely unknown; however, previous studies have demonstrated that RSV at micromolar range modulates multiple molecular targets associated with inflammation and immunity [56]. Accordingly, we treated our samples with a small concentration of RSV (i.e., 10 μM), creating a condition that reflects a physiological situation; indeed, although the properties of RSV have been widely investigated “in vitro” using high concentrations (50–200 μM), these elevated levels are never reached in vivo [57], as only low micromolar concentrations are achievable in human biological fluids with a daily intake of extra-virgin olive oil [58], or after a single oral dose of RSV [59].

Although clinical studies on humans are lacking, the results from in vitro models have shown the efficacy of RSV in ameliorating redox status, protecting lymphocytes and enhancing cytotoxicity when used in combination with anticancer drugs [60]. In our study, the increased cytotoxic activity seemed not to be attributable to the antioxidant activity of RSV, as TAC was comparable in activated lymphocytes in the absence or presence of RSV. Nevertheless, the possibility that the beneficial effects on the killing phase could be ascribed, at least in part, to the capacity of RSV to bind and modulate estrogen receptor signaling cannot be ruled out. It is well known that aging and chronic deficiency of sex hormones may have important effects on the immunity of menopausal women [61] with consequences on the health status that can be partially controlled by a healthy lifestyle [62,63,64]. Indeed, following menopause, women are increasingly vulnerable to common NCDs, and several studies have found a correlation between the age of menopause onset and the risk of NCDs [17,65,66]. On the contrary, hormone replacement therapy partially reverses menopause-related immunological changes reducing the pro-inflammatory cytokine production and improving NK cell activity [67]. As RSV is structurally similar to estrogens and binds equally to the α and β estrogen receptors subtypes [44], based on our results, we speculate that RSV may act on cytotoxic lymphocytes by modulating estrogen-dependent process signaling pathways. Nevertheless, this hypothesis needs more investigation. In this regard, the limitation of this study is represented by the lack of age-matched men and fertile women samples. To verify this hypothesis, a different experimental design, including control populations and specific biological approaches, is needed to demonstrate the estrogenic mechanism of action at the genomic and non-genomic levels.

## 5. Conclusions

Recent preclinical studies have indicated RSV as a promising support against NCDs owing to its estrogenic, anti-inflammatory, and antioxidant properties. Our study provides additional evidence of the potential beneficial effect of RSV on the immune surveillance, demonstrating that it may increase the cytotoxic potential against malignant cells in menopausal women, probably due to its affinity for the estrogen receptor. These results designate RSV as a useful supplement capable of ameliorating the immune function of menopausal women. While the possible dimorphic effect of RSV on lymphocyte cytotoxicity needs further investigation and additional efforts to be demonstrated, its ability to optimize NK cell-mediated immunity may have potential applications in nutraceutical and menopause research.

## Data Availability

The data presented in this study are available in article.
